# Rapid Genetic Diagnosis for Okinawan Patients with Enlarged Vestibular Aqueduct Using Single-Stranded Tag Hybridization Chromatographic Printed-Array Strip

**DOI:** 10.3390/jcm11041099

**Published:** 2022-02-19

**Authors:** Akira Ganaha, Eiji Hishinuma, Tadashi Kaname, Masahiro Hiratsuka, Shunsuke Kondo, Tetsuya Tono

**Affiliations:** 1 Department of Otorhinolaryngology-Head and Neck Surgery, University of Miyazaki, Miyazaki 889-1692, Japan; tono@med.miyazaki-u.ac.jp; 2 Advanced Research Center for Innovations in Next-Generation Medicine, Tohoku University, Sendai 980-8573, Japan; ehishi@ingem.oas.tohoku.ac.jp (E.H.); masahiro.hiratsuka.a8@tohoku.ac.jp (M.H.); 3 Tohoku Medical Megabank Organization, Tohoku University, Sendai 980-8573, Japan; 4 Department of Genome Medicine, National Center for Child Health and Development, Tokyo 157-8535, Japan; kaname-t@ncchd.go.jp; 5 Laboratory of Pharmacotherapy of Life-Style Related Diseases, Graduate School of Pharmaceutical Sciences, Tohoku University, Sendai 980-8578, Japan; 6 Department of Pharmaceutical Sciences, Tohoku University Hospital, Sendai 980-8574, Japan; 7 Department of Otorhinolaryngology-Head and Neck Surgery, University of the Ryukyus, Okinawa 903-0215, Japan; h082544@med.u-ryukyu.ac.jp

**Keywords:** Pendred syndrome, enlarged vestibular aqueduct, *SLC26A4*, rapid diagnosis, Okinawan patients

## Abstract

Both Pendred syndrome (PS) and nonsyndromic hearing loss with an enlarged vestibular aqueduct (EVA) are autosomal recessive disorders caused by *SLC26A4* pathogenic variants. The spectrum of *SLC26A4* pathogenic variants varies with the ethnic background. Among the patients with EVA in Okinawa, 94% had some combination of NM_000441.2(SLC26A4):c.1707+5G>A and NM_000441.2(SLC26A4):c.2168A>G(p.His723Arg), the two *SLC26A4* pathogenic variants that are the most common in this population. We identified these two pathogenic variants using a novel genotyping method that employed an allele-specific polymerase chain reaction (PCR) from a gDNA and single-stranded tag hybridization chromatographic printed-array strip (STH-PAS) in DNA samples obtained from 48 samples in Okinawa, including 34 patients with EVA and 14 carriers of c.1707+5G>A or c.2168A>G. In addition, whole blood and saliva samples were used for analysis in this genotyping method with direct PCR. The results of STH-PAS genotyping were consistent with those obtained using standard Sanger sequencing for all samples. The accuracy of the STH-PAS method is 100% under the optimized conditions. STH-PAS genotyping provided a diagnosis in 30 out of 34 patients (88%) in Okinawan patients with EVA in under 3 h. The turn-around time for STH-PAS genotyping used with direct PCR was 2 h as a result of the omission of the DNA extraction and purification steps. Using information about the ethnic distribution of pathogenic variants in the *SLC26A4* gene, STH-PAS genotyping performs a rapid genetic diagnosis that is simple and has a considerably improved efficiency.

## 1. Introduction

**Figure 1:**
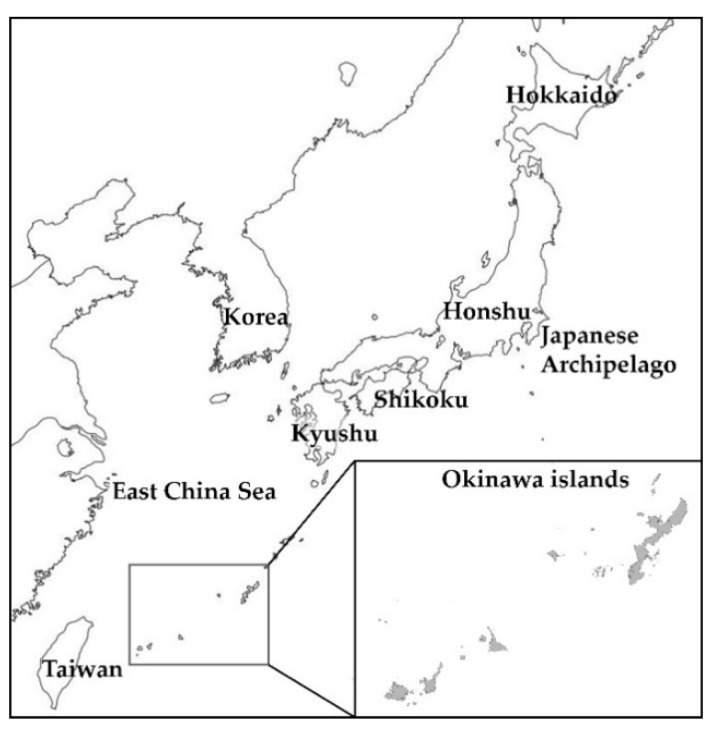
Geographic location of the Okinawa Islands: this string of small islands is situated between the Japanese island of Kyushu and Taiwan.

An enlarged vestibular aqueduct (EVA) is a common inner ear malformation in patients with hearing loss that is frequently associated with Pendred syndrome (PS) [1,2]. PS is characterized by congenital sensorineural hearing loss with EVA and goiter [3]. Both nonsyndromic hearing loss with EVA and PS are autosomal recessive disorders caused by pathogenic variants in the *SLC26A4* gene [4]. *SLC26A4* is the second-most common gene associated with deafness among Japanese patients [5]. A difference in the ethnic distribution of the spectrum of pathogenic variants in *SLC26A4* was revealed in previous studies [6,7]. Whereas only approximately 25% of Caucasian patients with EVA were identified with two pathogenic variants in *SLC26A4* [8,9,10], this fraction is much higher, at 67–90%, in the Asian population [11,12,13,14,15]. In Japan, NM_000441.2(SLC26A4):c.2168A>G(p.His723Arg) (Table 1) is the most common *SLC26A4* pathogenic variant, associated with 36.0% of patients. The second is NM_000441.2(SLC26A4):c.919-2A>G, with 7.0%, followed by NM_000441.2(SLC26A4):c.1707+5G>A (Table 1), with 4.0% of patients [12]. The Okinawa Islands are the southwestern-most islands of the Japanese archipelago (Figure 1), whose population carries a different spectrum of *SLC26A4* pathogenic variants than other populations [16]. One notable feature of this genetic difference is the *SLC26A4* pathogenic variant c.1707+5G>A (Table 1), which is the most common pathogenic variant uniquely reported in patients with EVA in the Okinawa Islands [16]. Previous genome-wide single-nucleotide polymorphisms (SNPs) data indicate a difference in clusters between mainland Japan (Hondo cluster) and the Okinawa Islands (Ryukyu cluster) [17]. According to a publicly accessible whole genome reference database of 14,000 healthy Japanese individuals [18], the allele frequencies of the c.1707+5G>A and c.2186A>G pathogenic variants are 0.00014 and 0.00205, respectively. Thus, in the general population of Japan, and in contrast to the Okinawans, the allele frequency of the c.1707+5G>A pathogenic variant is much lower than that of c.2186A>G. This suggests that the high frequency of the c.1707+5G>A pathogenic variant is due to a founder effect [16].

To date, the various genotyping methods for detecting point mutations, including Sanger sequencing, polymerase chain reaction–restriction fragment length polymorphism (PCR-RFLP), allele specific PCR, TaqMan real-time PCR, invader assay and microarray, have been used [19,20,21,22,23]. These techniques require expensive instrumentation and technical expertise for reliable outcomes [24]. Recently, a genotyping method was developed that employs allele-specific PCR and a single-stranded tag hybridization chromatographic printed-array strip (STH-PAS) [25]. STH-PAS genotyping does not require electrophoresis or DNA sequencing, providing the results in under 15 min after allele-specific PCR. This rapid and efficient method is capable of a safe and high-sensitivity visualization of multiplex DNA signals [24,25].

Herein, we report a protocol that uses allele-specific PCR and STH-PAS and is optimized to genotypically identify the c.1707+5G>A and c.2168A>G pathogenic variants, the two most common pathogenic variants of *SLC26A4* in patients with EVA in the Okinawa Islands. In addition, we evaluated the feasibility of a novel genotyping method that applies direct PCR and STH-PAS to whole blood and saliva samples.

## 2. Materials and Methods

### 2.1. Subjects

The DNA samples of a total of 48 Okinawan samples were analyzed. All patients were diagnosed with hearing loss in the Department of Otorhinolaryngology, Head and Neck Surgery of the University of the Ryukyus, Japan. Of these, 34 were diagnosed with PS or EVA, and, by genotype, they included twelve with compound heterozygous pathogenic variants for c.1707+5G>A and c.2168A>G, nine with homozygous pathogenic variant of c.1707+5G>A, nine with homozygous pathogenic variant for c.2168A>G, two with compound heterozygous pathogenic variant for c.2168A>G and some other *SLC26A4* pathogenic variants and two genetically undiagnosed (Table 2). In addition, 14 DNA samples were examined as a carrier of c.1707+5G>A or c.2168A>G, including three samples with heterozygous pathogenic variant for c.1707+5G>A and eleven with heterozygous pathogenic variant for c.2168A>G. For ground truth, prior Sanger sequencing confirmed c.1707+5G>A and/or c.2168A>G in *SLC26A4* in all samples. Whole blood and saliva samples from one Okinawan patient with compound heterozygous for c.1707+5G>A and c.2168A>G were examined by direct PCR and STH-PAS genotyping without nucleic acid extraction.

### 2.2. Primer Design

The sequence of primers used for PCR amplification is listed in Table 3. The forward primer was labeled with a tag-spacer sequence at its 5′ end for detection with STH-PAS. The nucleotide at the 3′ end of each forward primer was changed according to each mutation to be used for allele-specific PCR. Each reverse primer was labeled with biotin at its 5′ end. In the forward primers, except c.1707_Fw_Vt, the nucleotides immediately before the 3′ end were modified to become different sequences to avoid nonspecific amplification [26]. In the c.1707_Fw_Vt primer, we could not adopt the mismatched nucleotide at position −1 of its 3′ end because of low PCR amplification. Instead, we added an extra nucleotide, T, at position +1 of its 3′ end, to improve the PCR amplification.

### 2.3. PCR Amplification from gDNA

DNA was extracted using the Qiagen DNA Extraction Kit (Qiagen; Hilden, Germany) according to the manufacturer’s instructions and quantified using high-sensitivity spectrophotometry (Nano Drop 1000, Labtech Ltd.; East Sussex, UK).

Multiplex single-tube PCR amplification was performed using a 20 μL reaction mixture of 4 components, including (i) distilled water (6 µL); (ii) genomic DNA samples (1 µL; 10 ng/μL), (iii) 2× KAPA2G Fast Multiplex Mix (Kapa Biosystems; Massachusetts, USA) (10 μL) and (iv) primer mixture (3 μL; 5 μM), c.1707_Fw_Wt, c.1707_Fw_Vr, c.1707_Rv, c.2168_Fw_Wt, c.2168_Fw_Vr and c.2168_Rv.

PCR was performed under the following condition: (i) initial denaturation step (95 °C; 3 min) and (ii) 28 cycles of the 3-step (95 °C; 15 s) → (60 °C; 30 s) → (72 °C; 30 s) sequence in the PCR Thermal Cycler (GeneAlas G02, Astec, Fukuoka, Japan).

Stability of this protocol was confirmed with PCR performed at a higher (50 ng/uL) and lower (5 ng/uL) DNA concentration at different annealing temperatures (59–60 °C or 61 °C).

### 2.4. Direct PCR Amplification from Whole Blood and Saliva Samples

PCR using whole blood and saliva samples was performed in a direct sample processing method in which samples are added to the amplification reaction without prior extraction or quantification of DNA. Briefly, whole blood was collected into EDTA tubes, and saliva into FastGene^TM^ centrifuge tubes (Nippon Genetics, Tokyo, Japan). To each sample, 0.1 mL of DNAzol Direct reagent (Molecular Research Center Inc., Cincinnati, OH, USA) was mixed using the manufacturer’s protocol, and the mixture was incubated (80 °C; 10 min) before being directly subjected to PCR.

The amplification protocol was identical for both types of sample. The reactions were initially performed using a reaction mixture of 20 μL composed of 2 μL of the DNA template, prepared as described earlier. The PCR procedure followed a sequence of (i) initial denaturation step (95 °C; 3 min), followed by (ii) 31 cycles of the 3-step (95 °C; 15 s) → (60 °C; 30 s) → (72 °C; 30 s). The difference from the sequence used for gDNA amplification was 3 extra cycles.

### 2.5. Dipstick DNA Chromatography

**Figure 2:**
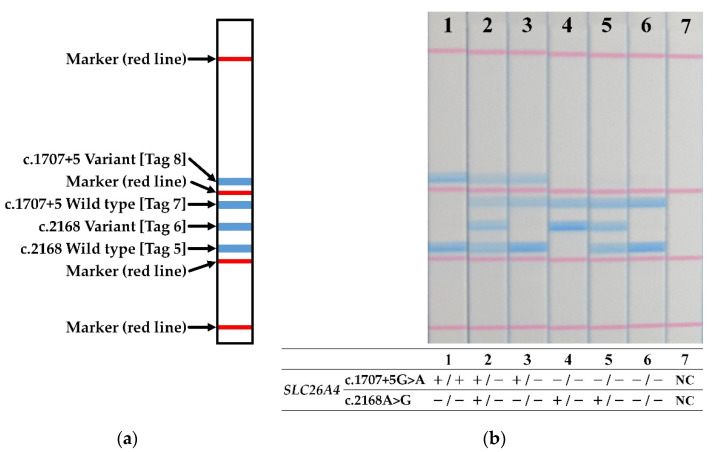
(**a**) Schematic diagram of the STH-PAS genetic analysis. (**b**) Results of PCR amplicon signals detected by STH-PAS for seven subjects that are representative of the entire group of patients. Red lines are positional markers. Blue lines indicate the presence of pathogenic variants. The genotype, represented by the pattern of blue lines, was summarized at the bottom of each lane. NC, negative control; −, template pathogenic variant absent; +, template pathogenic variant carrier.

Dipstick DNA chromatography printed array (C-PAS) strips and reagents were obtained commercially (TBA Co.; Sendai, Japan). STH-PAS genotyping was performed in a 20 µL mixture of 4 components, including (i) separation buffer (10 µL), (ii) distilled water (8 µL), (iii) streptavidin-coated blue latex suspension (1 µL) and (iv) the PCR product (1 µL). The C-PAS strip was dipped and held into this mixture (10 min at room temperature) to facilitate the reaction, in which, streptavidin-coated blue latex reacted to combine with the biotin at the 5′ end of the amplicons. Hybridization of each forward primer with the oligonucleotides complementary to their 5′ terminal tags, printed on lines at distinct position along the C-PAS membrane, appears as a visible blue line in a distinct position (Figure 2a). Essentially, the assay reveals different genotypes in *SLC26A4* in a “bar code” made of the blue lines. The diagnosis of the genotype for each sample was based on the pattern of blue lines on the STH-PAS (Figure 2a) made through visual inspection.

## 3. Results

We studied 34 patients who were clinically diagnosed with hearing loss and EVA. Table 2 summarizes the patients’ *SLC26A4* genotypes, as well as phenotypes of gender, affected side, vertigo, goiter and hearing level. No significant differences were expected in the distributions of phenotypes among the five genotype groups, owing to the small sample of 34 patients.

The genotypes of the 34 patients with Sanger-detected *SLC26A4* pathogenic variants were determined by the STH-PAS protocol described here using 10 ng/μL of their gDNA. Representative results from seven samples are shown in Figure 2b. No false positives or false negatives were identified. All of the results of 48 samples, including 34 patients and 14 carriers, were consistent with Sanger sequencing (Supplementary Figure S1).

**Figure 3:**
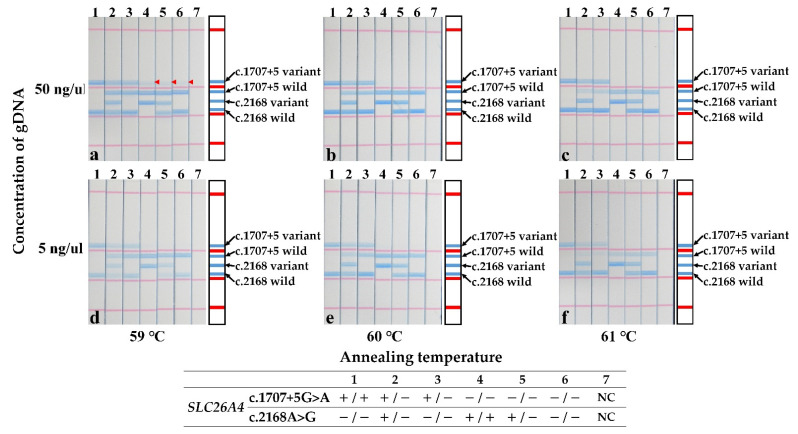
Stability test of the STH-PAS genetic analysis for the c.1707+5G>A and p.H723R pathogenic variants in *SLC26A4*. Each panel summarizes results obtained at a different pair of conditions. Rows are organized by template DNA concentration: top row (**a**–**c**), high (50 ng/μL); bottom row (**d**–**f**), low (5 ng/μL). Similarly, columns are organized by annealing temperature: left (**a**,**d**), 59 °C; middle (**b**,**e**), 60 °C; and right (**c**,**f**), 61 °C. Red arrowheads (strips 4, 5, 6 in **a**) indicate false positives. NC, negative control; −, template pathogenic variant absent; +, template pathogenic variant carrier.

To test the stability of the STH-PAS protocol, control experiments were conducted with two different concentrations of template DNA and three different annealing temperatures. The results are summarized in Figure 3. Except for the false-positive results observed at a high (50 ng/μL) DNA concentration at the annealing temperature of 59 °C (marked by red arrows in Figure 3a), the STH-PAS genotyping protocol worked without false positives or false negatives in a large concentration range of template DNA at annealing temperatures of 60–61 °C (Figure 3b–f).

**Figure 4:**
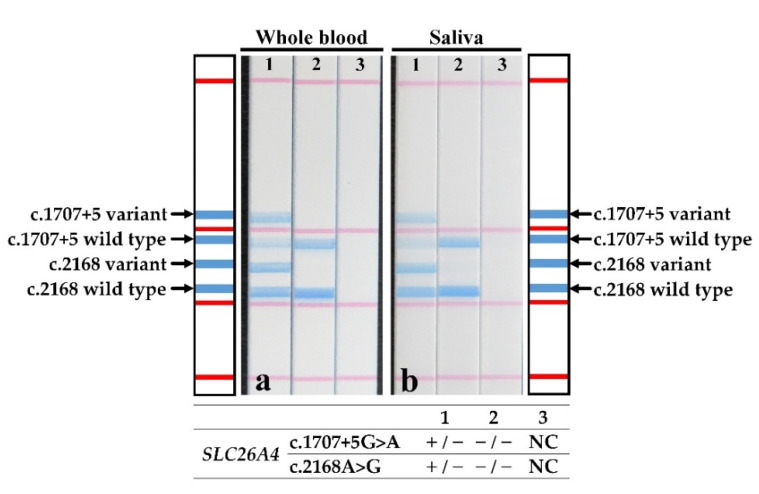
Results of the STH-PAS genetic analysis for c.1707+5G>A and p.H723R in *SLC26A4* using direct PCR from samples of (**a**) whole blood and (**b**) saliva of a single patient (strip 1) and two controls (strips 2 and 3). The genotype of each lane was presented in the bottom graphics. NC, negative control; −, template pathogenic variant absent; +, template pathogenic variant carrier.

Only one patient had both whole blood and saliva samples collected. The patient’s genotype was determined by the direct PCR and STH-PAS protocol described here, using whole blood (Figure 4a) and saliva (Figure 4b), in addition to the genotyping by PCR and STH-PAS using gDNA (Figure 2b, lane 2). The results of STH-PAS genotyping using direct PCR were consistent with those obtained using standard Sanger sequencing (Figure 4).

## 4. Discussion

With a focus on Okinawan patients with EVA, we developed a rapid genotyping method that targets *SLC26A4* pathogenic variants c.1707+5G>A and c.2168A>G using allele-specific PCR and STH-PAS. The STH-PAS genotyping does not involve electrophoresis and DNA sequencing, providing results within 3 h. Furthermore, the STH-PAS genotyping used with direct PCR provides results even faster, within 2 h, owing to the circumvention of the steps of DNA extraction and purification. Interpreting the results of genotyping is as simple as reading off bar codes. The sample preprocessing required for STH-PAS, including DNA extraction and PCR amplification, costs approximately USED 7–8 per sample [24]. Thus, by saving effort and time, the STH-PAS method represents an improvement over alternative genotyping assays used to detect specific genetic variations. In addition, STH-PAS is safe because the PCR amplicons are visualized as a blue line on the C-PAS without relying on ethidium bromide, a chemical identified as a safety risk with carcinogenic effects [27,28].

Apart from the clear advantages of STH-PAS genotyping, there is a definite limit to this method. There are, at most, twelve test lines currently available on the C-PAS, and four lines were used in this study; this is sufficient to detect the presence or absence of no more than six pathogenic variants. Thus, with current C-PAS strips, the STH-PAS genotyping method is not useful when the genetic diagnosis requires the identification of more than six variants.

Ensuring the robustness of a rapid assay is essential. It was reported that STH-PAS genotyping had a 100% sensitivity and specificity [24,25]. Correct DNA-template binding, i.e., restricting binding only to the template with perfect complement, is a highly temperature-sensitive step in the PCR protocol, balancing the trade-off between two objectives. A low annealing temperature leads to a lower primer specificity binding with a template DNA [29]. Meanwhile, high annealing temperature leads to a weakly amplified reaction [29]. Thus, to ensure that we operated at the optimized annealing temperature, multiplex single-tube PCR amplification was performed, probing different annealing temperatures in a narrow range (59–61 °C). Based on our results, the annealing temperature in the multiplex PCR should be set to 60 °C; the method used at lower temperatures could produce false positives.

To avoid the nonspecific annealing of primers, another parameter, the number of PCR amplification cycles, was also adjusted, and 26–30 was identified as the adequate range. The method was less sensitive to DNA concentrations, working well under low concentrations and producing accurate results in a broad range (5 to 50 ng/μL). Tian et al. also reported that PCR-dipstick DNA chromatography could detect quantities of DNA amplicons as low as 100 pg and could demonstrate a higher sensitivity than PCR-agarose gel electrophoresis [25]. Thus, DNA chromatography has a high sensitivity and the optimization of the PCR protocol is critical for obtaining accurate results.

Performing PCR directly on whole blood or saliva samples can help to circumvent the extraction of gDNA from the sample, which has substantial benefits in terms of the time required and effort expended [30]. This simplified approach also minimizes sample-to-sample contamination despite skipping multiple steps usually required for manual extraction [30]. Saliva is a viable DNA source for genotyping studies [31], and, unlike methods collecting blood samples, saliva sampling is non-invasive and does not require trained medical personnel. Furthermore, saliva presents a lower risk of infection than blood [31]. We showed that the accuracy of genotyping by STH-PAS used with direct PCR from whole blood and saliva samples was comparable to that obtained by Sanger sequencing of DNA extracted from blood. Thus, coupling the STH-PAS genotyping method with direct PCR from whole blood or saliva is a feasible, safe and non-invasive option, presenting multiple benefits in clinical practice.

The ethnicity-specific STH-PAS genotyping based on the distribution of pathogenic variants in *SLC26A4* improves the efficiency of a genetic diagnosis in patients with EVA. At most, the presence or absence of six pathogenic variants could be identified with a single C-PAS membrane. Whereas identification of the most frequent six *SLC26A4* pathogenic variants allows for a genetic diagnosis of 94% (32/34) in Okinawan patients in this study, the diagnostic rate of the same approach is estimated to vary from 34–73% in East Asian patients with EVA, given the prevalence of pathogenic variants in *SLC26A4* previously revealed in these populations [12,14,32]. Thus, an STH-PAS genotyping method appropriately tailored to the genetic variations in targeted ethnicities could be applied broadly in East Asia. Meanwhile, it is difficult to apply the STH-PAS genotyping for diagnosis in Caucasians because only approximately 25% of Caucasian patients with EVA were identified with two pathogenic variants in *SLC26A4* [8,9,10].

One limitation of our study is its design, targeting only c.1707+5G>A and c.2168A>G, the two most common *SLC26A4* pathogenic variants in Okinawan patients with EVA. Another one is the small sample size overall; and the third one is the sample-keeping procedure, which limited us to one patient and one control in order to evaluate the combined direct PCR and STH-PAS test. Thus, a further study using a larger number of patients with EVA is necessary to firmly establish the accuracy of our novel rapid genotyping method.

## 5. Conclusions

We developed a multiplex variant detection method by combining allele-specific PCR with STH-PAS to verify the presence or absence of the two *SLC26A4* pathogenic variants, c.1707+5G>A and c.2168A>G, that are the most common among Okinawan patients with hearing loss and EVA. Furthermore, by combining direct PCR and STH-PAS from whole blood and saliva specimens, we developed a novel, rapid and less invasive genotyping method. The diagnostic accuracy of STH-PAS genotyping for the two pathogenic variants was 88% correct in Okinawan patients with EVA. The STH-PAS genotyping method appropriately tailored to the genetic variations in targeted ethnicities could be applied broadly in other geographical regions.

## Figures and Tables

**Table 1:** Details of the target pathogenic variants.

	NM_000441.2(SLC26A4):c.2168A>G(p.His723Arg)	NM_000441.2(SLC26A4):c.1707+5G>A
Position	chr7:107710132	chr7:107700180
Reference SNP number	rs121908362	rs192366176
Nucleotide	NC_000007.13:g.107350577A>G	NC_000007.13:g.107340625G>A
Functional consequence	Loss of function variant	Sequence variant affecting splicing

**Table 2:** Distribution of *SLC26A4* genotypes and phenotypes in 34 patients.

Genotypes	Patients	Gender (M: F)	Bilateral: Unilateral	Vertigo	Goiter	Threshold (dB)	
						Right	Left
c.1707+5G>A homozygote	9 (26.5%)	3: 6	9: 0	6 (67%)	6 (67%)	96.6	100.0
c.2168A>G homozygote	9 (26.5%)	4: 5	9: 0	5 (56%)	4 (44%)	98.2	91.3
c.1707+5G>A/c.2168A>G	12 (35.3%)	4: 8	11: 1	7 (58%)	6 (50%)	95.6	101.5
c.2168A>G/c.1579A>C	1 (2.9%)	0: 1	1: 0	1	1	91.7	71.7
c.2168A>G/c.1229C>T	1 (2.9%)	1: 0	1: 0	1	0	101.7	95.0
Undiagnosed	2 (5.9%)	0: 2	0: 2	0	0	57.0	57.0
Total	34	12: 22	31: 3	20	18	90.1	86.1

**Table 3:** Allele-specific PCR primers.

Variant	Primer Name	Primer Sequence (5′–3′)	Size (bp)
c.2168A>G	c.2168_Fw_Wt	[Tag 5]-spacer- GGACACATTCTTTTTGACGGTCGA	219
	c.2168_Fw_Vt	[Tag 6]-spacer- GGACACATTCTTTTTGACGGTCTG	
	c.2168_Rv	[Biotin]-TGAGGCTCCATGAAGTTATATAGG	
c.1707+5G>A	c.1707_Fw_Wt	[Tag 7]-spacer- AATGTATCAAGTCCACAGTAGG	361
	c.1707_Fw_Vt	[Tag 8]-spacer- AATGTATCAAGTCCACAGTAAAT	
	c.1707_Rv	[Biotin]-GCACAAAGCCTGTTAGTCCA	

## Data Availability

All data generated or analyzed during this study are included in this published article.

## Supplementary Materials

The supporting information can be downloaded at https://www.mdpi.com/article/10.3390/jcm11041099/s1: Figure S1: Results of the STH-PAS genetic analysis in all 48 samples for c.1707+5G>A and p.H723R in *SLC26A4*.
