# Supplementary material for: Rapid Genetic Diagnosis for Okinawan Patients with Enlarged Vestibular Aqueduct Using Single-Stranded Tag Hybridization Chromatographic Printed-Array Strip
Source: J Clin Med. 2022 Feb 19;11(4):1099. doi: 10.3390/jcm11041099 (PMC8880462; doi:10.3390/jcm11041099)
Supplement: Supplementary file 1 [file jcm-11-01099-s001.zip › jcm-1582651-supplementary.pdf]

Supplementary Material

Ganaha A et al. **Rapid Genetic Diagnosis for Okinawan Patients with Enlarged Vestibular Aqueduct Using Single-stranded Tag Hybridization Chromatographic Printed-array Strip.** *J. Clin. Med.* **2022**, *11*, 1099. www.mdpi.com/article/10.3390/jcm11041099/s1

Contents

Figure S1.

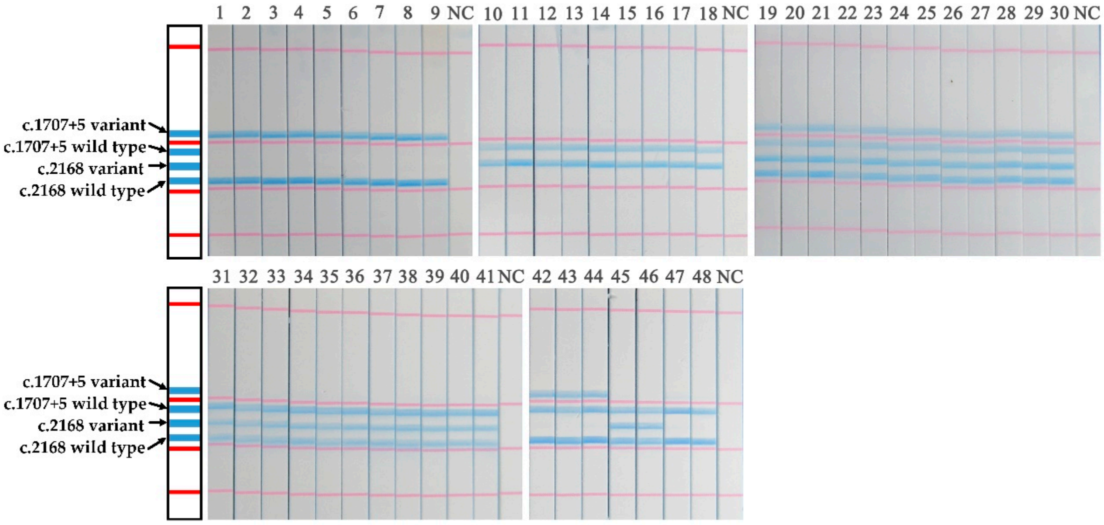

Figure S1.

Genetic analysis of 48 samples for the c.1707+5G>A and p.H723R mutations in the SLC26A4 gene. Single-stranded tag hybridization chromatographic printed-array strip (STH-PAS) genotyping method was used with 10 ng/μL DNA concentration at the annealing temperature of 60°C. No false positives or false negatives were identified.

1–9: c.1707+5G>A homozygotes;

10–18: c.2168G>A homozygotes;

19–30: c.1707+5G>A + c.2168G>A compound heterozygotes;

31–41: c.2168A>G heterozygotes;

42–44: c.1707+5G>A heterozygotes;

45: c.2168A>G + c.1579A>C compound heterozygote;

46: c.2168A>G + c.1229C>T compound heterozygote;

47–48: no mutation identified in *SLC26A4*;

NC: negative control.
